# A three-dimensional morpho-volumetric similarity study of Down syndrome keratopathy vs. keratoconus

**DOI:** 10.1186/s40662-022-00315-0

**Published:** 2023-01-03

**Authors:** Ibrahim Toprak, Francisco Cavas, José S. Velázquez, Jorge L. Alio del Barrio, Jorge L. Alio

**Affiliations:** 1 Department of Ophthalmology, Faculty of Medicine, Pamukkale University, Denizli, Turkey; 2 Bioengineering and Applied Computational Simulation Research Group, Technical University of Cartagena, Cartagena, Spain; 3 Department of Structures, Construction and Graphical Expression, Technical University of Cartagena, Cartagena, Spain; 4 Department of Research and Development, VISSUM, Calle Cabañal, 1, Edificio Vissum, 03016 Alicante, Spain; 5 Cornea, Cataract and Refractive Surgery Department, VISSUM, Calle Cabañal, 1, Edificio Vissum, 03016 Alicante, Spain; 6 Division of Ophthalmology, Department of Pathology and Surgery, Faculty of Medicine, Miguel Hernández University, Calle Cabañal, 1, Edificio Vissum, 03016 Alicante, Spain

**Keywords:** Corneal model, Corneal structure, Down syndrome, keratoconus, Keratopathy, Morphogeometry

## Abstract

**Background:**

To compare and contrast morpho-volumetric features between Down syndrome (DS) cornea and non-DS keratoconic cornea by three-dimensional (3D) modelling.

**Methods:**

Forty-three subjects (43 eyes) with DS and 99 patients matching their age and sex (99 eyes) with keratoconus (KC) were included in this single-center cross-sectional study. Main outcome measures were high-order aberrations (HOA), central corneal thickness (CCT), spherical equivalent refraction, and morpho-volumetric parameters established using a 3D corneal model, such as deviation of anterior and posterior corneal apices (D_apexant_/D_apexpost_) and minimum thickness points (D_mctant_/D_mctpost_) from corneal vertex, areas of the anterior and posterior surfaces (A_ant_/A_post_), sagittal area passing through the anterior and posterior corneal apices (A_apexant_/A_apexpost_) and minimum thickness point (A_mctpost_) and corneal volume of the complete cornea (V_total_).

**Results:**

Age, gender, spherical equivalent refraction, CCT and V_total_ were similar between the net on-DS KC and DS groups (*P* > 0.05), while non-DS KC group had higher HOA than the DS group (*P* < 0.05). D_apexant_, A_ant_, A_post_ and A_apexant_ showed higher values in the DS group than in the non-DS KC group, whereas D_apexpost_ showed a reduction in the DS group when compared with the non-DS KC group (*P* < 0.05).

**Conclusions:**

This study demonstrated that anterior and posterior corneal apex dynamics were specifically different in DS subjects, as the anterior apex tends to displace more prominently when compared to that from the non-DS KC group, while the posterior apex appears to be more stable than that in non-DS KC, which also support the theory that DS patients suffer from a specific keratopathy, distinctively different to KC but strongly related to it, and probably showing a diversity of corneal phenotypes in all cases of DS.

## Background

Down syndrome (DS), also known as trisomy 21, is characterized by cognitive impairment and multisystemic disorders, as well ocular abnormalities [1].

Incidentally, there is a well-established relationship between DS and the occurrence of keratoconus (KC) [1–3]. Alió et al. recently showed that 71.3% of DS patients had topographical anomalies in their corneas that were consistent with KC [4]. Furthermore, several investigations also revealed that individuals with DS had steeper, thinner, and more aberrated corneas, even though these alterations did not fulfil the diagnostic criteria for KC [4–7].

In the current literature, the majority of previous investigations relied on topographic and pachymetric features of the cornea in DS [4, 5, 7–10]. However, a recent study by Toprak et al. first revealed three-dimensional (3D) features of the cornea in a DS population [11]. They demonstrated that the anterior corneal apex was displaced in the DS group even with normal topography, while the posterior apex tended to be more stable although topography was abnormal when compared to the non-DS individuals with normal corneal topography [11]. They utilized a novel 3D virtual corneal model previously introduced by Cavas et al. that offers the advantage to integrate the topographic data from both corneal surfaces with the tomography profile of the whole central 8 mm cornea [12–14]. This method assumes that alterations in corneal collagen structure and/or organization affect 3D morphogeometric and volumetric parameters, and this notion has been validated in the diagnosis of subclinical and clinical KC [12–15].

Based on the understanding that individuals with DS show distinct corneal characteristics that were inherently abnormal, and many times are comparable to or identical with those in eyes with KC, the goal of this study is to evaluate with a more global perspective 3D morphogeometric and volumetric aspects of the cornea in a DS group vs. a non-DS group with KC to investigate if there is a DS-related keratopathy that varies from non-DS KC. Moreover, this original study also aims to test the potential similarities between KC and DS corneas analyzed for the first time with this new morpho-volumetric analysis which offers a wider and more integrated perspective on corneal analysis.

## Methods

The principles of the Declaration of Helsinki for the research with human subjects involved were followed, and the study was approved by the Institutional Ethics Committee (CEI21-001). VISSUM Innovation (Cornea, Cataract and Refractive Surgery Unit, Alicante, Spain), Keratoconus IBERIA databases (Universidad Miguel Hernández de Elche, Elche, Spain) and Bioengineering and Applied Computational Simulation Research Group databases (Universidad Politécnica de Cartagena, Cartagena, Spain) contributed data for the study.

This retrospective non-randomized cross-sectional study was conducted at VISSUM Innovation and comprised consecutive 99 non-DS subjects with KC (99 eyes) and age- and sex- matched 43 DS patients (43 eyes) with genotypic confirmation, who were examined between 2017 and 2019.

One eye was selected for statistical analysis in bilateral cases using randomization function of the Statistical Package for Social Sciences (SPSS) version 24 software (IBM SPSS Statistics Inc., Chicago, IL, USA). Eyes with history of surgery, hydrops, corneal trauma, scarring, infection, and unacceptable quality of the topographic test (score < 90%) were excluded.

All participants underwent a detailed ophthalmological examination and anterior segment tomography (Sirius System^®^, CSO, Firenze, Italy). Topography measurements were performed by a single experienced optometrist for three times for each eye and the one with the highest coverage and centration scores over 90% with a green checkmark was used for further analysis.

Contact lens wearers were requested to remove their contact lenses for 2 (for soft lens) and 4 weeks (for hard lens) prior to the measurements.

Two cornea specialists performed clinical and topographical examinations (JLA and JADB). To avoid bias, the Sirius System^®^ topographic classifier outputs (as “Normal,“ “KC suspect,“ “KC compatible,“ and “Abnormal or treated”) were validated by two observers with consensus. Following criteria were used to confirm the KC diagnosis; presence of typical biomicroscopic and retinoscopic signs of KC (if any) such as Rizzuti’s phenomenon, Fleischer’s ring, scissoring, Vogt’s striae, Munson’s sign and and/or any typical pattern for KC on axial/tangential curvature map [superior steep, inferior steep, irregular, oval, round, inferior-steep and superior-steep asymmetric bowtie, symmetric or asymmetric bowtie with skewed radial axes (SRAX) > 22 degrees and inferior-superior (I-S) keratometric asymmetry ≥ 1.5 D] and coexistence of central/paracentral or inferior focal steepening (anterior and/or posterior) with corresponding corneal thickness reduction. All measurements were performed following the standard operative procedures defined by the EVICR.net for the corneal topography examination and performed by personnel certified in Good Clinical Practices.

The Sirius System^®^ classifier report was also considered; borderline topographical alterations were considered “KC suspect” when they did not comply with the above-mentioned KC criteria. Topographical findings that did not match any sort of corneal ectasia pattern were considered “Abnormal or treated”. Topographical maps those lacked any of the above-mentioned abnormalities were defined as “Normal”.

Based on topographical categorization, the overall DS group was categorized into two subcategories: DS with KC topography (eyes with topographic classification of “KC compatible” and “KC suspect”), and DS with non-KC topography (eyes with topographic classification of “Abnormal or treated” and “Normal”).

The non-DS and DS groups were compared in terms of age, gender, spherical equivalent, central corneal thickness (CCT), high-order corneal aberrations (HOA), and 3D morphogeometric and volumetric parameters.

### Patient-specific 3D corneal modelling & morphogeometric parameters

The patient-specific 3D corneal model used to determine the morphogeometric parameters used in this study was generated directly from the raw data acquired by the Sirius tomographer (clouds of topographical points that define anterior and posterior corneal surfaces), following a procedure created by Cavas and colleagues [14], that has been validated and has also already been applied to the diagnosis of KC [16, 17], to the detection of subclinical keratoconus [15] and to the study of the evolution of invasive treatments on corneal ectasias [18] in several previous studies.

**Fig. 1:**
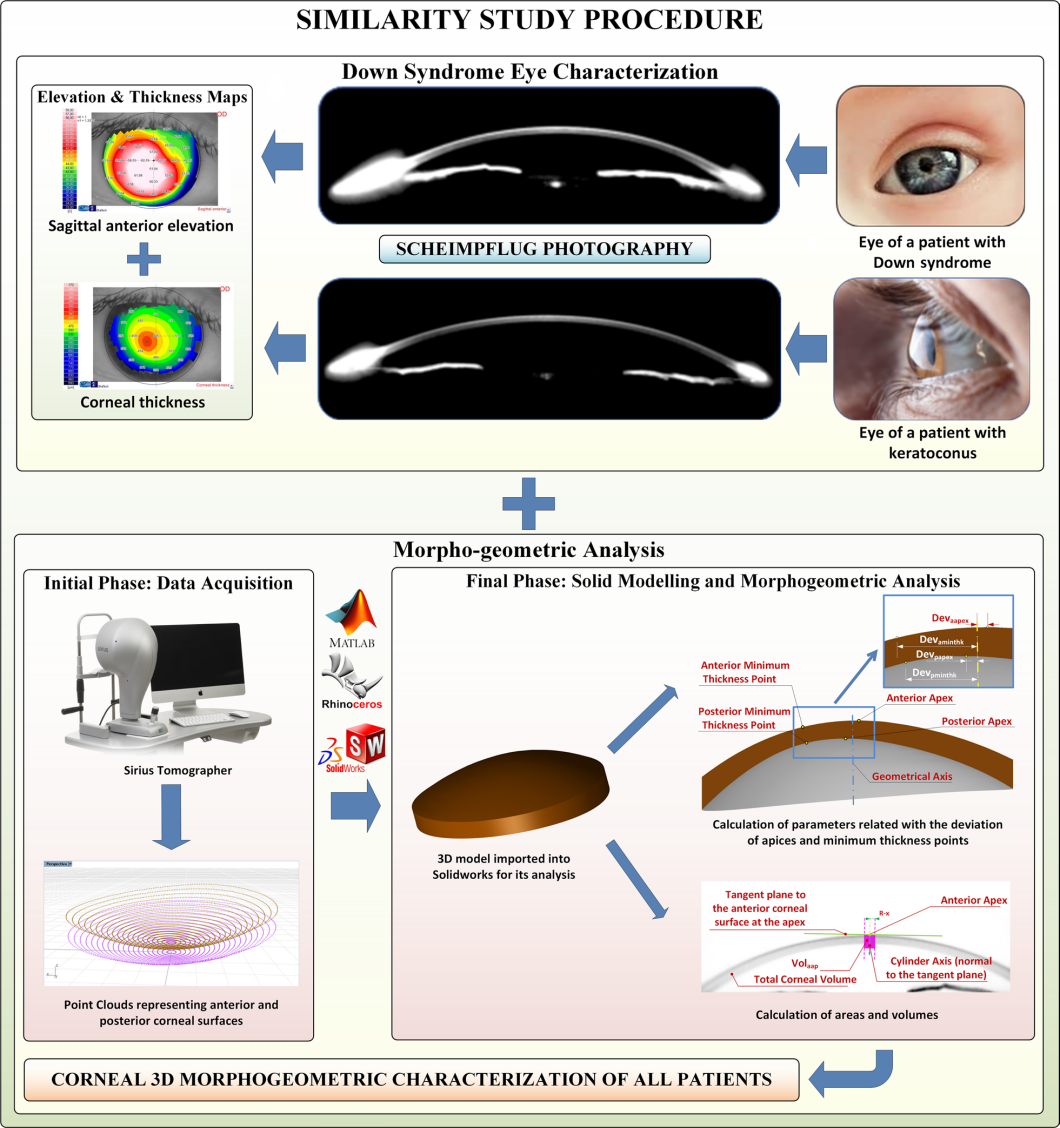
Scheme of the procedure used to study the similarity between Down syndrome (DS) and non-DS corneas

The procedure consists of two well-differentiated phases (Fig. 1): an initial one in which the tomographical data is obtained, followed by a final one in which 3D model is generated and a morphogeometric analysis is made to determine several linear, surface, volumetric and/or angular parameters. A detailed description of both the procedure and the definition of the main morphogeometric parameters can be found in [14].

### Statistical analysis

A minimum sample of 40 eyes per group was needed (DS and non-DS KC groups) at 95% power and 95% confidence level with an effect size (d) of 0.78 (GPower v0.3.1.9.6, Universität Düsseldorf, Düsseldorf, Germany).

After completing the study, statistical power was re-calculated based on the comparisons among non-DS KC group (n = 99), DS with KC topography (n = 16) and DS with non-KC topography (n = 27) groups and was found to be 90.6% (GPower v0.3.1.9.6, Universität Düsseldorf, Düsseldorf, Germany).

SPSS version 24 (IBM SPSS Statistics Inc., Chicago, IL, USA) was the software selected for statistical analysis. The Chi-square test was selected for comparison purposes with qualitative data (gender) between groups. The Kolmogorov-Smirnov test was conducted to ensure that the variables had a normal distribution. Age, volumetric, morphogeometric, aberrometric, pachymetric and refractive data were represented as mean ± standard deviation (SD). When the parametric test assumptions were fulfilled, a t-test for independent samples (Student’s t-test, two-tailed) was used for quantitative data comparison purposes between both non-DS and DS groups. The Mann-Whitney U test (two-tailed) was utilized in all other cases. At the 95% confidence interval, a *P* value less than 0.05 was considered statistically significant.

## Results

There were no differences regarding age, gender, spherical equivalent refractive error, and CCT between the non-DS KC group (n = 99) and the DS group (n = 43) (*P* > 0.05, Student’s t-test and Mann-Whitney U test, Table 1), whereas HOA was significantly higher in the non-DS KC group than in the DS group (*P* < 0.05, Mann-Whitney U test, Table 1). In the DS group, 16/43 (37.2%) eyes showed KC-related topography, which were classified as “KC compatible” (n = 7), “KC suspect” (n = 9), and 27/43 (62.8%) eyes had non-KC topography with topographic classification of “Normal” (n = 18) and “Abnormal or treated” (n = 9). The non-DS KC group and DS with KC topography groups were matched regarding the KC severity (all had the RETICS stage III KC) to eliminate statistical bias.

**Table 1:** Comparison of the non-Down syndrome (DS) keratoconus (KC) group and DS group with regards to age, refractive, aberrometric, pachymetric and morpho-volumetric data

Variables (mean ± SD)	Non-DS KC group (*n* = 99)	Down syndrome group total (*n* = 43)	*P*
Age (years)	27.1 ± 6.9	24.3 ± 11.3	0.145^a^
Spherical equivalent (diopters)	− 1.99 ± 3.05	− 0.70 ± 4.52	0.098^b^
HOA RMS (µm)	**1.44 ± 0.79**	**1.22 ± 1.10**	**0.004**^**b**^
Central corneal thickness (µm)	500.6 ± 31.2	500.8 ± 29.5	0.453^a^
D_apexant _(mm)	**0.003 ± 0.006**	**0.007 ± 0.010**	**< 0.0001**^**b**^
D_apexpost _(mm)	**0.161 ± 0.074**	**0.076 ± 0.048**	**< 0.0001**^**b**^
D_mctant_(mm)	1.045 ± 0.322	1.453 ± 1.042	0.400^b^
D_mctpost _(mm)	0.975 ± 0.305	1.368 ± 1.015	0.425^b^
A_ant _(mm^2^)	**43.19 ± 0.18**	**43.46 ± 0.25**	**< 0.0001**^**a**^
A_post_ (mm^2^)	**44.39 ± 0.30**	**44.60 ± 0.42**	**0.018**^***b***^
A_tot_ (mm^2^)	103.22 ± 1.18	103.33 ± 1.51	0.641^a^
A_apexant _(mm^2^)	**1.64 ± 1.98**	**3.98 ± 0.29**	**< 0.0001**^**a**^
A_apexpost_ (mm^2^)	3.99 ± 0.26	3.97 ± 0.27	0.625^a^
A_mctpost _(mm^2^)	3.98 ± 0.26	3.94 ± 0.28	0.400^a^
C_x _(mm)	**0.016 ± 0.043**	− **0.004 ± 0.048**	**0.012**^**a**^
C_y _(mm)	0.026 ± 0.035	0.020 ± 0.041	0.408^b^
C_z _(mm)	**0.763 ± 0.027**	**0.784 ± 0.029**	**0.001**^**b**^
V_total_	23.95 ± 1.53	23.60 ± 1.82	0.239^a^

*A*_*ant*_, *A*_*post*_ = area of the anterior and posterior corneal surfaces; *A*_*tot*_ = sum of anterior, posterior and perimetric corneal surface areas; A_apexant_, *A*_*apexpost*_ = sagittal plane apex area of the cornea within the sagittal plane passing through the Z axis and the highest point (apex) of the anterior and posterior corneal surfaces; *A*_*mctppost*_ = sagittal plane area of the cornea within the sagittal plane passing through the Z axis and the minimum thickness points in the posterior corneal surfaces; *C*_*x*_, *C*_*y*_, *C*_*z*_ = center of mass coordinates (X, Y, Z) of the solid; *D*_*apexant*_, *D*_*apexpost*_ = average distance from the Z axis to the highest point (apex) of the anterior and posterior corneal surfaces; *D*_*mctant*_, *D*_*mctpost*_ = average distance in the XY plane from the Z axis to the minimum thickness points of the anterior and posterior corneal surfaces; *DS* = Down syndrome; *HOA* = high-order aberrations; *KC* = keratoconus; *RMS* = root mean square; *SD* = standard deviation

*P* < 0.05 and bold values indicate statistical significance

^a^ Student’s t-test, *P* < 0.05 indicates statistically significant difference

^b^ Mann-Whitney U test, *P* < 0.05 indicates statistical significance

Regarding morpho-volumetric parameters, the DS group had significantly higher deviation of anterior corneal apices (D_apexant_), area of the anterior surface (A_ant_), area of the posterior surface (A_post_), anterior corneal apices (A_apexant_) and center of mass coordinates (Z) of the solid (C_z_) values when compared to those from the non-DS KC group, whereas D_apexpost_ and center of mass coordinates (X) of the solid (C_x_), were significantly lower in the DS group than in the non-DS KC group (*P* < 0.05, Student’s t-test and Mann-Whitney U test, Table 1). On the other hand, corneal volume of the complete cornea (V_total_) did not differ between the DS and non-DS KC groups (*P* > 0.05, Student’s t-test, Table 1).

Comparing non-DS KC group, DS with KC topography and DS with non-KC topography subgroups, age, spherical equivalent refractive error (absolute value) and HOA were higher in the non-DS KC group than in the DS with non-KC topography subgroup (*P* < 0.05, Table 2). In both DS subgroups, D_apexant,_ A_ant_ and A_apexant_ were higher, and D_apexpost_ was lower when compared to those from the non-DS KC group (*P* < 0.05, Table 2). The DS with KC topography subgroup represented lower C_x_ and higher C_z_ values than in the non-DS KC group (*P* < 0.05, Table 2). The A_apexpost_ and posterior corneal minimum thickness point (A_mctpost_) were found to be higher in the DS with non-KC topography subgroup compared to those from the DS with KC topography subgroup (*P* < 0.05, Table 2). In the DS with KC topography subgroup, center of mass coordinates (Y) of the solid (C_y_) and V_total_ were significantly lower than in the non-DS KC group and DS with non-KC topography subgroup (*P* < 0.05, Table 2).

**Table 2:** Comparative analysis of age, refractive, aberrometric, pachymetric and morpho-volumetric data among the non-Down syndrome (DS) keratoconus (KC) group, DS with non-KC topography and DS with KC topography groups

Variables (mean ± SD)	Non–DS KC group (*n* = 99)	DS non–KC topography (*n* = 27)	DS KC topography total (*n* = 16)	Comparison of three groups P*	Statistically significant pairwise comparisons P**
Age (years)	27.1 ± 6.9	22.3 ± 10.1	27.6 ± 12.7	0.034	(0.039)^a^
Spherical equivalent (diopters)	− 1.99 ± 3.05	− 0.18 ± 4.56	− 1.65 ± 4.44	< 0.0001	(< 0.0001)^a^
HOA RMS (µm)	1.44 ± 0.79	1.02 ± 0.89	1.55 ± 1.35	0.003	(0.002)^a^
Central corneal thickness (µm)	500.6 ± 31.2	505.3 ± 23.7	479.0 ± 34.1	0.059	*P* > 0.05
D_apexant_ (mm)	0.003 ± 0.006	0.004 ± 0.008	0.011 ± 0.011	< 0.0001	(0.022)^a^, (< 0.0001)^b^
D_apexpost_ (mm)	0.161 ± 0.074	0.061 ± 0.033	0.100 ± 0.060	< 0.0001	(< 0.0001)^a^, (0.003)^b^
D_mctant_ (mm)	1.045 ± 0.322	1.467 ± 1.036	1.428 ± 1.086	0.620	*P* > 0.05
D_mctpost_ (mm)	0.975 ± 0.305	1.384 ± 1.021	1.341 ± 1.037	0.646	*P* > 0.05
A_ant_ (mm^2^)	43.19 ± 0.18	43.43 ± 0.22	43.50 ± 0.31	< 0.0001	(< 0.0001 both)^a,b^
A_post_ (mm^2^)	44.39 ± 0.30	44.60 ± 0.38	44.59 ± 0.51	0.054	*P* > 0.05
A_tot_ (mm^2^)	103.22 ± 1.18	103.64 ± 1.40	102.80 ± 1.60	0.301	*P* > 0.05
A_apexant_ (mm^2^)	1.64 ± 1.98	4.06 ± 0.25	3.83 ± 0.32	< 0.0001	(< 0.0001)^a^, (0.009)^b^
A_apexpost_ (mm^2^)	3.99 ± 0.26	4.05 ± 0.24	3.83 ± 0.27	0.035	(0.029)^c^
A_mctpost_ (mm^2^)	3.98 ± 0.26	4.02 ± 0.24	3.80 ± 0.29	0.016	(0.013)^c^
C_x_ (mm)	0.0160 ± 0.0430	− 0.0009 ± 0.0390	− 0.009 ± 0.061	0.014	(0.035)^b^
C_y_ (mm)	0.026 ± 0.035	0.034 ± 0.022	− 0.002 ± 0.055	0.027	(0.044)^b^, (0.031)^c^
C_z_ (mm)	0.763 ± 0.027	0.788 ± 0.025	0.777 ± 0.035	0.001	(0.001)^b^
V_total_	23.95 ± 1.53	24.12 ± 1.62	22.73 ± 1.85	0.015	(0.027)^b^, (0.017)^c^

*A*_*ant*_, *A*_*post*_ = area of the anterior and posterior corneal surfaces; *A*_*tot*_ = sum of anterior, posterior and perimetric corneal surface areas; *A*_*apexant*_, *A*_*apexpost*_ = sagittal plane apex area of the cornea within the sagittal plane passing through the Z axis and the highest point (apex) of the anterior and posterior corneal surfaces; *A*_*mctppost*_ = sagittal plane area of the cornea within the sagittal plane passing through the Z axis and the minimum thickness points in the posterior corneal surfaces; *C*_*x*_, *C*_*y*_, *C*_*z*_ = center of mass coordinates (X, Y, Z) of the solid; *D*_*apexant*_, *D*_*apexpost*_ = average distance from the Z axis to the highest point (apex) of the anterior and posterior corneal surfaces; *D*_*mctant*_, *D*_*mctpost*_ = average distance in the XY plane from the Z axis to the minimum thickness points of the anterior and posterior corneal surfaces; *DS* = Down syndrome; *HOA* = high-order aberrations;  *KC* = keratoconus; *RMS* = root mean square; *SD* = standard deviation

*P* < 0.05 and bold values indicate statistical significance

*Comparison of the non-DS KC group, DS with non-KC topography and DS with KC topography subgroups (independent samples Kruskal-Wallis test, *P* < 0.05 indicates statistically significant difference)

**Pairwise comparisons among the three groups (independent samples Kruskal-Wallis test, adjusted *P* < 0.05 indicates statistically significant difference after post-hoc corrections)

^a^Statistically significant difference between the non-DS KC group and DS with non-KC topography group

^b^Statistically significant difference between the Non-DS KC group and Down syndrome with KC topography group

^c^Statistically significant difference between the DS with non-KC topography and DS with KC topography group

## Discussion

The current study revealed that subjects with DS had similar CCT, and the thinnest point deviation (D_mct_) to those from non-DS KC patients. However, in the DS group, regardless of whether they had KC topography or not, deviation from corneal vertex was greater for the anterior corneal apex (D_apexant_) and lower for the posterior apex (D_apexpost_) when compared to those from the non-DS KC group. These 3D morpho-volumetric findings can be valuable for explaining the specific KC-like topographical and pachymetric features of the cornea in DS population, which were also previously suggested by the topography-based clinical studies [4, 6, 7, 10].

Our research group previously evaluated morphogeometric features of the cornea in non-DS KC and showed that anterior and posterior corneal apex and the thinnest point significantly deviated from corneal vertex in varying stages of KC in non-DS population [12–15]. Furthermore, a recent study by our group demonstrated that anterior apex and the thinnest point showed significant deviation from corneal vertex in DS patients when compared to the non-DS controls with normal topography, while posterior corneal apex displacement was similar between the DS and normal non-DS subjects [11]. Considering the results of the above-mentioned studies and our current study, the anterior and posterior apex dynamics seem to be different via morpho-volumetric analysis in DS subjects; the anterior apex tends to displace more prominently when compared to that from both non-DS control and non-DS KC groups while the posterior apex seems to be more stable than that seen in non-DS KC.

On the other hand, the present study also revealed that DS corneas had similar corneal thickness and the thinnest point deviation to those seen in eyes with KC in a non-DS population.

Although decreased corneal thickness and volume in DS corneas were also previously reported by several topography-based studies [8–10], this study demonstrated more significant corneal volume reduction in DS subjects with KC when compared with those from the stage-matched non-DS KC and DS without KC groups.

In the current study, the DS group had larger anterior and posterior surface areas (A_ant_ and A_post_) as well as anterior sagittal area at the corneal apex (A_apexant_). The existence of anterior and posterior corneal surface irregularities such as localized elevations increasing the surface area might explain these observations.

Regarding center of mass coordinates of the cornea, the DS group had higher z value and lower x value than in the non-DS KC group, whereas y value was similar between the DS and non-DS KC groups. This is due to the fact that the geometrical point that dynamically represent translation manifests a more prolate behavior in the DS group than in the non-DS KC group, and therefore a corneal volume reduction takes place for the DS group [19]. These results are in tandem with other studies [5, 9], but to our knowledge, it is the first time that the results are validated in a patient-specific 3D morpho-volumetric study.

The relatively small sample size in the DS group can be considered as a limitation of this study. However, despite the modest sample size in the DS group, it was considered to be strong enough to support the conclusions as far as calculated statistical power of the study was 90.6%.

## Conclusion

In conclusion, the findings of this morphogeometric study aid to understand previous clinical studies showing corneal topographical similarities between a non-DS keratoconic cornea and a DS cornea. However, beyond the topographical evidence, this study is the first to demonstrate that anterior and posterior corneal apex dynamics were especially different in DS subjects and non-DS subjects, further demonstrating that DS corneas have a different topographical and morphovolumetric profile from non-DS corneas. This reinforces the theory already raised by our group that corneal structure and anatomy are specific features of all DS cases [4], predisposing them to a number of corneal pathologies that influence the stability of their corneas which consequently challenges their quality of vision along their lives. These findings might have resulted from similarities and differences in corneal microstructure and biomechanics between DS cornea and non-DS KC and support the theory that DS patients suffer from a specific keratopathy, distinctively different to KC but strongly related to it. The structure and organization of corneal collagen may potentially be compromised by DS, as multi-systemic collagen abnormalities are not uncommon in DS patients. However, further investigations into the ultrastructure of the anterior and posterior stromal collagen network in the DS population may support our theory. Lastly, DS cases offer a diversity of corneal phenotypes that could be possible to identify in a larger number of cases. The finding of such phenotypes and their potential for further evolution is the subject of ongoing investigation in our research group.

## Data Availability

The data presented in this study are available on request from the corresponding author. The data are not publicly available due to privacy reasons.
